# The Influence of Work Environment Factors on the Ocular Surface in a One-Year Follow-Up Prospective Clinical Study

**DOI:** 10.3390/diagnostics11030392

**Published:** 2021-02-25

**Authors:** Edyta Chlasta-Twardzik, Aleksandra Górecka-Nitoń, Anna Nowińska, Edward Wylęgała

**Affiliations:** 1 Chair and Clinical Department of Ophthalmology, Faculty of Medical Sciences in Zabrze, Medical University of Silesia, 40-055 Katowice, Poland; a_gorecka@op.pl (A.G.-N.); anna.nowinska@sum.edu.pl (A.N.); wylegala@gmail.com (E.W.); 2 Ophthalmology Department, Railway Hospital, 40-760 Katowice, Poland

**Keywords:** dry eye disease, ocular surface, work environment factors, noninvasive keratograph break-up time

## Abstract

The purpose of this study was to assess the effect of environmental working conditions on the symptoms and signs of dry eye disease and to examine whether and how those conditions impact the ocular surface. Methods: This single-center, prospective clinical study with a 1-year follow-up included 150 patients. The following parameters were evaluated: non-invasive keratograph break-up time (NIKBUT), tear meniscus height (TMH), and conjunctival and limbal hyperemia. We also performed staining of the surface of the eye for simulated fluorescein images, Schirmer’s test I, assessment of Meibomian gland dysfunction (MGD), and an Ocular Surface Disease Index (OSDI) questionnaire. Results: In the OW (office workers) group, in people working >4 h at the computer, the NIKBUT before work and the Schirmer test results were statistically significantly lower than in people working <4 h. The conjunctival hyperemia result before work was statistically significantly higher for people working >4 h at a computer in both groups and after work in the MW (medical workers) group. Low relative air humidity in the building and air-conditioned rooms negatively affects the tear film, causing the symptoms of dry eye disease. At the 1-year follow-up, there was a statistically significant reduction in conjunctival and limbal hyperemia in the OW group as well as a statistically significant reduction in TMH at the first examination before and after work, and in the second examination after 1 year in both groups. Conclusions: Environmental factors such as reduced relative air humidity, increased air temperature, and decreased illumination have a negative impact on the ocular surface.

## 1. Introduction

Dry eye disease (DED) is a common ocular surface disorder. Its complexity was highlighted by the updated definition in the recent official report of the International Dry Eye Workshop (DEWS 2017): “Dry eye is a multifactorial disease of the ocular surface characterized by a loss of homeostasis of the tear film, and accompanied by ocular symptoms, in which tear film instability and hyperosmolarity, ocular surface inflammation and damage, and neurosensory abnormalities play etiological roles” [1]. In a recent clinical trial, Laihia et al. demonstrated that disruption of hyperosmolar stress is indispensable for rescue processes on the ocular epithelia to begin. This can be achieved by supporting the stability of the tear film layers, normalizing its osmolarity and establishing continuous biophysical protection for the ocular surface [2]. The prevalence of DED has been reported in many countries around the world, with a range of between 5% and 50% [3,4,5]. The significant difference in prevalence of DED has been suggested to be influenced by geographical location, variations in study populations, as well as variations in the diagnostic criteria used, with an observed lack of standard diagnostic criteria, poor standardization of the groups of patients, nonstandardized questionnaires, and a lack of objective tests and diagnostic criteria. There are reports in the literature that the prevalence of DED in Asian countries is greater than in Western countries [6]. The prevalence of DED increases significantly with age and is more prevalent among women, especially after menopause [7,8]. Established risk factors that may influence the occurrence or intensification of DED have been reported in the literature. Those include environmental factors such as extreme temperature or reduced relative humidity [7,9]. Smoking, contact lens wear, use of video display terminals (VDT) or monitors, and refractive surgery such as LASIK (laser-assisted in situ keratomileusis) are also additional risk factors associated with DED [10,11,12,13,14]. It has been proven in numerous studies that DED also occurs with anxiety disorders, sleep disorders, and depression [15,16]. Uptake of certain medications such as antihistamines [17], beta-blockers [18], and oral contraceptives [19] can also have an impact on the occurrence of DED. The symptoms of DED include ocular discomfort, dryness, grittiness, redness, foreign-body sensation, visual disturbance, and blurry vision, which can cause considerable discomfort in daily activities including reading, watching TV, using a computer, or working, and can result in major impairment of everyday life [20,21]. DED is diagnosed on the basis of a combination of sign and symptoms according to the TFOS (Tear Film and Ocular Surface Society) diagnostic test battery, although several studies have reported a poor correlation between DED signs and symptoms [22,23,24]. Keratograph 5M is a noninvasive corneal topographer able to investigate different parameters of the ocular surface, including noninvasive tear film breakup time, tear meniscus height, infrared meibography, lipid layer thickness, ocular redness, and conjunctival hyperemia. Thus, it provides a comprehensive noninvasive evaluation of the ocular surface. The objective repeatability and reproducibility of the parameters evaluated by the Keratograph 5M allows us to obtain reliable results, and thus it is essential as a diagnostic tool or in the follow-up of interventions or ocular surface alterations [25,26]. Although numerous studies have been conducted to investigate the nature of DED, there are scarce data about the characteristics of DED in healthcare personnel [25,26,27]. To the best of our knowledge, there has been no report on the nature of DED comparing medical and office workers in the same hospital workplace in Poland. Moreover, this is the first clinical study research to analyze eye surface parameters using reproducible, objective, noninvasive Keratograph 5M. Undoubtedly, an advantage of this research is the introduction of an objective, repeatable measuring tool that allows us to obtain reliable results next to the subjective assessment of DED with the use of Ocular Surface Disease Index (OSDI) questionnaire in one study. The goal of our study was to assess the effect of indoor conditions/environmental factors in the workplace on the symptoms and signs of dry eye disease and examine whether and how those conditions impact the ocular surface. Therefore, in the present study, we evaluated the influence of external and environmental factors on selected parameters of the eye surface in hospital employees.

## 2. Materials and Methods

### 2.1. Study Population

This single-center, prospective clinical study was carried out in the Clinical Department of Ophthalmology of Medical University of Silesia, District Railway Hospital in Katowice, Poland. The study was conducted in accordance with the Declaration of Helsinki. The study protocol was approved by the local Bioethical Commission at the Silesian Medical University in Katowice, Poland (Resolution No. KNW/0022/KB1/126/2016 of 25/10/2016). In accordance with the requirements of the Declaration of Helsinki, we informed the participants of the study about the purpose, nature, and method of the research being carried out. Then, after expressing written, informed consent, they were qualified for the research project. We included a total of 150 patients. Inclusion criteria included signing an informed consent form to participate in the study. Exclusion criteria included active ocular surface inflammation, infectious conjunctivitis or keratitis, allergic conjunctivitis, seasonal allergy, glaucoma, use of contact lenses or lubricating drops, connective tissue disorders, history of ocular trauma, history of ocular surgery including refractive surgery, systemic vasculitis, use of general medications (with a proven effect on the eye surface according to the TFOS DEWS II Epidemiology Report [7]), or a systemic disease that is proven to affect the ocular surface (TFOS DEWS II pathophysiology report; see Table 5, page 453) [27]. Only workers who fully understood the nature of the survey and agreed to participate were included in the study. Patients enrolled in the study were assigned to two groups. Group 1, OW (office workers), consisted of 75 persons (including 67 women and 8 men; mean age: 47 years). They were office workers, employees of the administration of the district railway hospital in Katowice, Poland. Group 2, MW (medical workers), also consisted of 75 persons (including 63 women and 12 men, mean age 47.1 years). They were department doctors and nurses (not working in the operating theater, without exposure to X-rays) of the district railway hospital in Katowice, Poland. In both groups there were participants who declared that they spent more or less than 4 h a day at a computer. Both groups had participants who worked in rooms with and without air conditioning. The participants underwent an examination before starting work and after work on the same day. The follow-up period was one year. After a one-year follow-up, all tests were repeated in all participants enrolled in the study.

### 2.2. Examinations

The clinical examination of each person was divided into the history and physical examination. Each of the study participants was asked to complete the OSDI (Ocular Surface Disease Index) questionnaire in the morning before starting a workday and after one year at the follow-up visit, as well as in the morning before starting a workday. The physical examination consisted of measuring the eye surface parameters twice during the working day (before starting work and after its completion) in each study group. Working time in the OW and MW groups was 8 h a day. The physical examination included automatic measurement of the first time of tear film breakage called NIKBUT (non-invasive keratograph break-up time), measurement of the meniscus tear height (mm) TMH (tear meniscus height), and automatic classification of conjunctival bulbar redness using the Jenvis Grading Scale. Automatic measurements were made with the use of an Oculus Keratograph 5M Topographer (Oculus Optikgeräte GmbH, Wetzlar, Germany). Additionally, the following examinations were conducted: staining the surface of the eye, simulated fluorescein images of the measured eye, Schirmer’s test I without anesthesia (Schirmer Tear Test; Optitech; Prayagraj, India), and assessment of Meibomian gland dysfunction (MGD) grade on the basis of two scales: the quality scale of secretions and discharge (secretion grade) and the Meibomian gland patency scale (expressibility grade). All measurements were performed in both eyes of each patient by 1 examiner. We examined the differences in ocular surface parameters between both eyes and their correlations. There were no statistically significant differences between the eyes. The automatic measurement may produce distorted results for the other eye; therefore, in our results we included only the first eye examined [28].

Automatic measurement of the first tear film break-up time NIKBUT (non-invasive keratograph break-up time) represents the measured time in seconds between the full opening of the eyelids after a complete blink and the first break in the tear film. Each NIKBUT measurement was carried out 3 times on each eye, with a minimum interval of 2 min between each measurement, in order to ensure that the tear film stabilized again after each measurement. A single measurement of the meniscus tear height (mm) TMH (tear meniscus height) was taken in the middle of the lower eyelid in the projection of the center of the pupil while the patient’s eyes were fixed on the central point. Conjunctival bulbar redness was graded on the nasal and temporal conjunctiva in 0.1 steps, with use of the Jenvis Grading Scale (grade 0–4; 0 for no redness and 4 for a maximum level of redness). Schirmer’s test I was performed without anesthesia using a sterile Schirmer strip 5 × 35 mm standard blotting paper strips (Tear Touch, New Delhi, India) that was placed at the junction of the lateral one-third to medial two-thirds of the lower lid and left for 5 min while the patient blinked normally. The length of wetted Schirmer strip was recorded in millimeters. We read the measurement result after 5 min. Tests were performed in a nonventilated, moderately lit room with no glare sources in the patient’s field of vision. The test was always performed under the same conditions.

Assessment of Meibomian gland dysfunction grade (MGD grade) was based on 2 scales: the quality scale of secretions and discharge (secretion grade) and the Meibomian gland patency scale (expressibility grade). The quantitative and qualitative evaluation of Meibomian gland secretions was made using a slit lamp by squeezing a small amount of secretion from several glands and assessing it. “In accordance with the recommendations of the MGD Workshop (2011), MILD MGD is indicated by a secretion grade 4–7, an expressibility grade of 1 and an amorphous/colour fringe lipid pattern. MODERATE MGD is indicated by meibomian gland orifice plugging, lid margin vascularity, a secretion grade 8–12, an expressibility grade of 2 and a meshwork or wave (flow) lipid pattern. SEVERE MGD is indicated by lid margin meibomian gland orifice drop-out or displacement, a secretion grade >13, an expressibility grade of 3 and an absent, globular or abnormal colour fringe lipid pattern” [29].

The criteria for dry eye syndrome diagnosis were as recommended by TFOS, except for tear film osmolarity testing. The accepted criteria for dry eye syndrome diagnosis for at least 1 eye are as follows: subjects who scored ≥13 points in the OSDI questionnaire and the NIKBUT tear film break time < 10 s or abnormal ocular surface staining result (staining of the eye surface with a fluorescein strip (Fluoro touch; 1 mg fluorescein sodium; Madhu Instruments, India) and lissamine green staining strip (Green Touch Strips; 1.5 mg Lisamine green; Madhu Instruments, New Delhi, India) or had >5 points defects of the corneal epithelium or >9 defects of the conjunctiva or epitheliopathy of the eyelid margin or = 2 mm long and or = 25% width) were classified as having dry eye syndrome.

In order to assess the water layer of the tear film, we measured the height of the tear meniscus (TMH) and performed the Schirmer test. In order to assess the pathophysiological causes of dry eye syndrome, we adopted the following criteria. For dry eye syndrome associated with the deficiency of the tear film water layer, the criterion of tear meniscus height TMH ≤ 0.2 mm was adopted; for the dry eye form associated with excessive tear film evaporation, the criterion of MGD was adopted; for the mixed form, both criteria were adopted—the presence of MGD and abnormal TMH ≤ 0.2 mm (MGD + THM ≤ 0.2 mm).

Objective examination of the working conditions of office and medical workers involved measurement of the temperature (°C) and relative air humidity (%) in medical personnel’s workplaces with the use of a Beurer HM 16 hygrometer and thermometer (air temperature measurement accuracy: +/−0.1 °C, humidity measurement range: 20–95%) on the day of the medical examination. Measurement of illumination in medical staff’s working rooms was performed using an Abatronic AB 8809A Luxmeter (Abatronic company, Radom, Poland). The measurement accuracy range was ± 3% rdg ± 0.5% f.s. (<10,000 Lx) ± 4% rdg ± 10 d (>10,000 Lx), performed on the day of the medical personnel examination. We also recorded the working hours of medical personnel and the presence of an air conditioning or ventilation system in the workplace. The average time spent at a screen was given by the respondents in the questionnaire.

### 2.3. Statistical Analysis

The statistical analysis was prepared using a Microsoft Excel 2007 spreadsheet, and this database was implemented in the R statistical program, v. 3.4.3. The analysis of quantitative variables (i.e., expressed in numbers) was performed by calculating the mean, standard deviation, median, quartiles, minimum, and maximum. The analysis of qualitative (i.e., non-numeric) variables was performed by calculating the number and percentage of occurrences of each value. The comparison of the values of qualitative variables in the groups was performed using the chi-squared test (with Yates’s correction for 2 × 2 tables) or Fisher’s exact test, where low expected frequencies appeared in the tables. A significance level of 0.05 was adopted in the analysis. The comparison of the values of quantitative variables in the 2 groups was performed using Student’s *t*-test (when the variable had a normal distribution in the analyzed groups) or the Mann-Whitney test (when the variable was not normally distributed). The comparison of the values of quantitative variables in 2 repeated measurements was performed using Student’s *t*-test for paired pairs (when the change in the value of the variable between measurements had a normal distribution) or Wilcoxon’s test for bonded pairs (when the distribution was different from the normal distribution). The influence of the quantitative variable on the dichotomous (two-state) variable was analyzed using logistic regression. Moreover, the multivariable analysis of the independent influence of many variables on the dichotomous (binary) variable was performed using the logistic regression method. The results are presented in the form of ORs (odds ratios) with a 95% confidence interval (CI). The normality of the distribution of variables was tested using the Shapiro-Wilk test.

## 3. Results

### 3.1. Participant Characteristics

A total of 150 participants enrolled in the study. All participants were examined before starting work and after finishing their work on a given day. The follow-up period for all groups was 1 year. At the 1-year follow-up, all tests were repeated in participants enrolled in the study. In the OW group, there were 63 participants. In the MW group, there were 65 participants. Some participants were excluded from the study after 1 year for the following reasons. In the OW group, three people started using eye drops due to the severity of eye discomfort, three people were diagnosed with systemic diseases, and six people did not report for the examination. In the MW group, two people started using eye drops due to the severity of eye discomfort, two people were diagnosed with systemic diseases, and six people did not report for the examination. The groups did not differ significantly in terms of gender or age. On the other hand, the groups differed statistically significantly (*p* < 0.05) in terms of the severity of DED and the sum of the points obtained on the basis of the OSDI questionnaire. The demographic characteristics of the study population are shown in Table 1.

**Figure 1:**
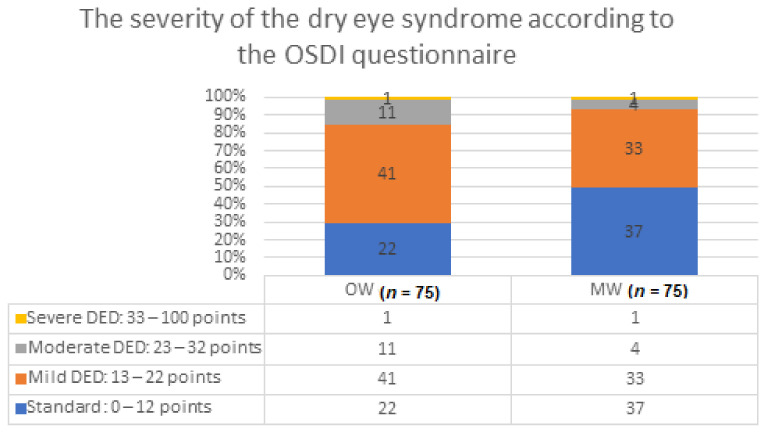
The severity of the dry eye syndrome according to the OSDI questionnaire. Legend: OW group—office workers group, MW group—medical workers group, DED—dry eye disease.

The severity of dry eye syndrome, on the basis of the points obtained in the OSDI questionnaire, in the OW and MW groups is presented in Figure 1. The sums of the points obtained differed statistically significantly (*p* < 0.05) between the groups. Subjective dry eye syndrome of mild (54.67%) and moderate severity (14.67%) was diagnosed in the OW group, which was significantly more frequent compared to the MW group.

### 3.2. Selected Parameters of the Eye Surface at Baseline on the First Day of the Examination

**Figure 2:**
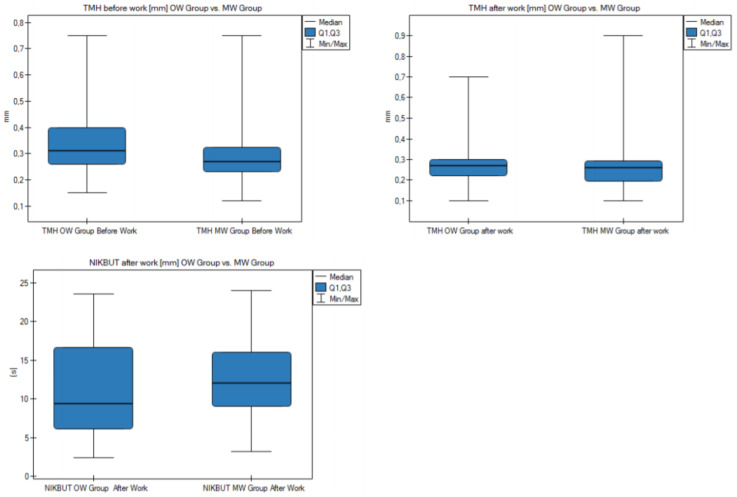
Comparison of the measurement results of the eye surface parameters in the OW and MW groups.

The groups differed statistically significantly (*p* < 0.05) in terms of the mean TMH result before and after work. In the OW group, it was 0.34 mm before work; in the MW group, it was 0.29 mm. The mean TMH result after work in the OW group was 0.29 mm, while in the MW group it was 0.26 mm. The difference of the mean NIKBUT result after work was statistically significant—in the OW group it was 11.18 s; in the MW group it was 12.53. Results are shown in Figure 2 and Table S1 (Table S1 is available in the Supplementary Materials).

**Figure 3:**
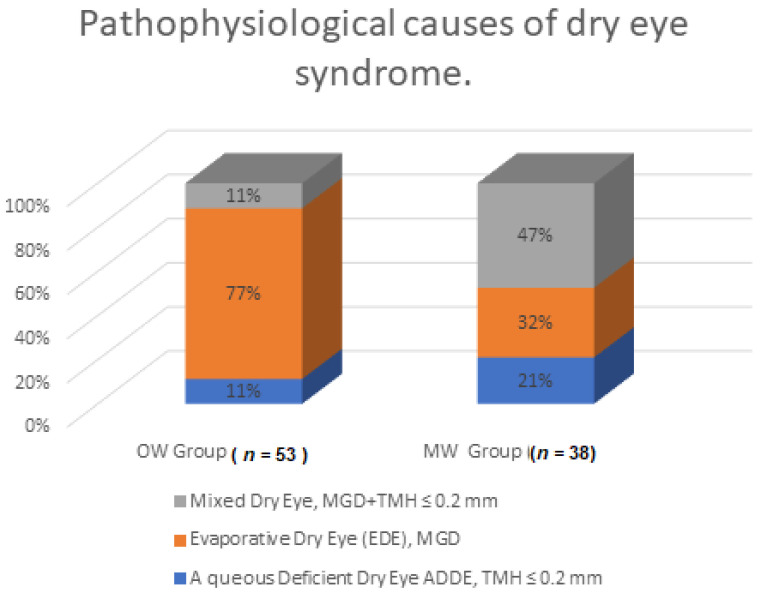
Pathophysiological causes of dry eye syndrome.

Figure 3 and Table 2 present the pathophysiological causes of dry eye syndrome. There was a statistically significant difference in the compared groups in terms of the pathophysiological cause of DED (*p* < 0.05). In the OW group, a significantly more frequent cause was evaporative dry eye and less frequent was aqueous deficient dry eye and mixed dry eye.

**Figure 4:**
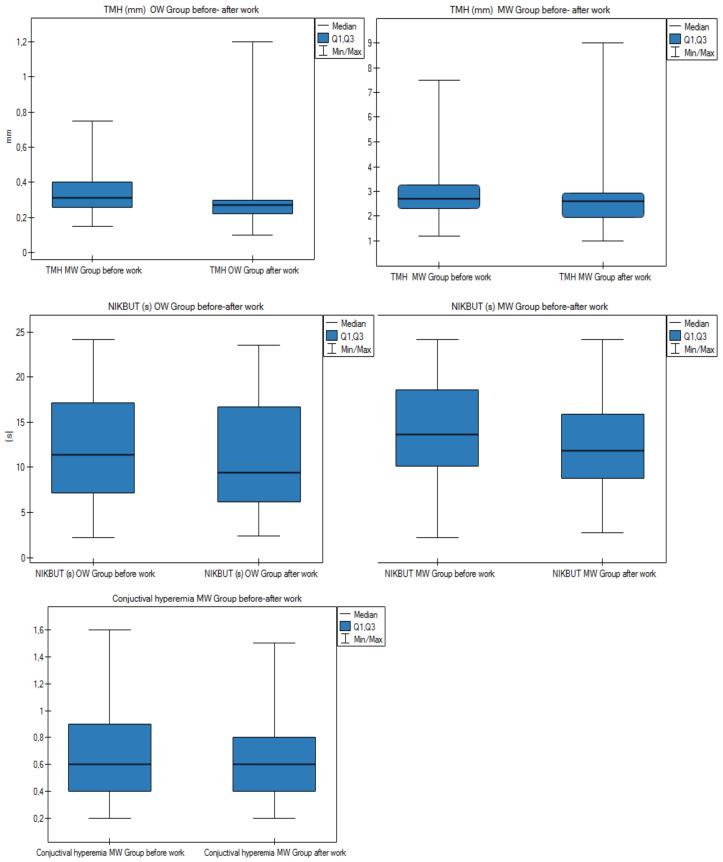
Measurement results of the eye surface parameters measured before and after work in the OW and MW groups. Table S2 is available in the Supplementary Materials.

To determine if there was a significant change in the measurements of the eye surface parameters, we made calculations for the before–after work differences in the OW and MW groups for the eyes’ examination. The results are presented in Figure 4 and Table S2 (Table S2 is available in the Supplementary Materials). In both the OW and MW groups, there was a statistically significant (*p* < 0.05) before–after work difference for TMH and NIKBUT. In the OW group, the mean TMH results were 0.34 before work and 0.29 after work, while in the MW group they were 0.29 before work and 0.26 after work. The mean NIKBUT results before and after work were 12.43 s vs. 11.18 s in the OW group and 14.11 s vs. 12.53 s in the MW group. Additionally, in the MW group, there was a statistically significant difference for the measurement of conjunctival hyperemia. The result was 0.71 before work and 0.67 after work. The results of measurements measured after work were lower than those before work.

### 3.3. Effect of Air Conditioning on the Ocular Surface

The influence of air conditioning on some parameters of the eye surface was demonstrated.

**Figure 5:**
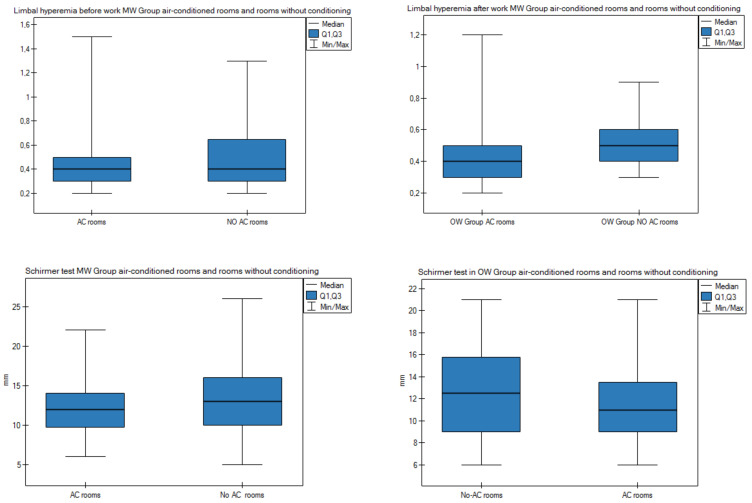
Comparison of the results of the parameters of the eye surface in the OW group and in the MW group in air-conditioned rooms and rooms without conditioning. Table S3 is available in the Supplementary Materials.

Figure 5 and Table S3 (Table S3 is available in the Supplementary Materials) present the parameters of the eye surface in the OW and MW groups for people who work in air-conditioned (AC) and non-air-conditioned (non-AC) rooms. Statistically significant differences were found in the following parameters: mean limbal hyperemia result before work (group MW: AC 0.54 vs. non-AC 0.47) and after work (group MW AC 0.53 vs. non-AC 0.487), and mean Schirmer test result for both groups (group OW AC 11.35 vs. non-AC 14.75; group MW AC 11.06 vs. non-AC 13.57).

### 3.4. Effect of Working Time at a Computer on the Ocular Surface

**Figure 6:**
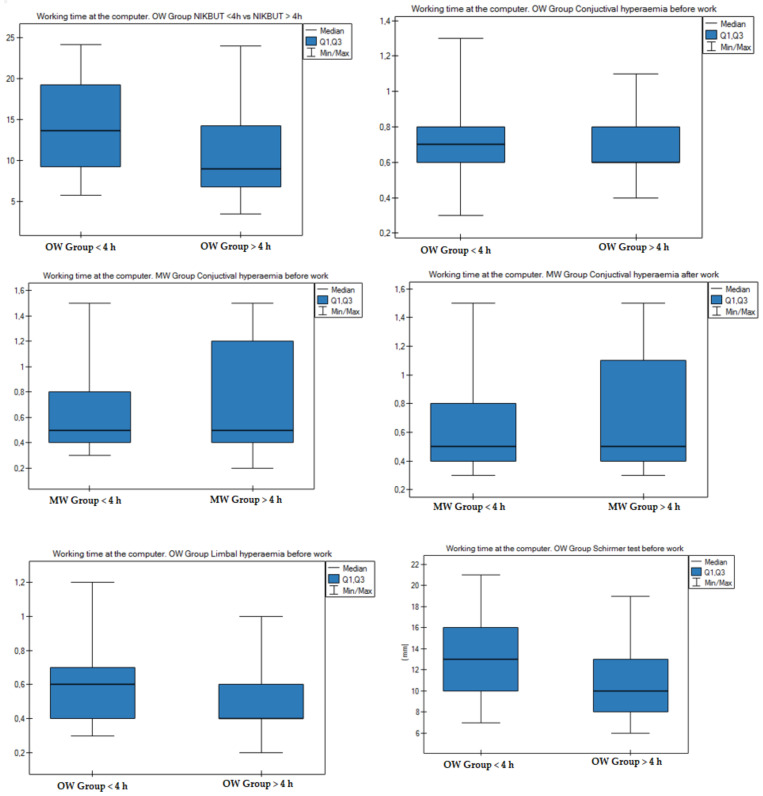
Working time at the computer and the results of measurements of the eye surface parameters in the OW and MW groups. Table S4 is available in the Supplementary Materials.

Figure 6 and Table S4 (Table S4 is available in the Supplementary Materials) present the eye surface parameters measured in the OW and MW groups for people who work at a computer for less than and more than 4 h. Statistically significant differences were found in the following parameters: mean NIKBUT result before work, mean conjunctival hyperemia result before work, mean conjunctival hyperemia result after work, mean limbal hyperemia result before work, and mean Schirmer test results.

To determine the risk of incorrect results of measurements of the eye surface parameters before and after work, as well as the influence of environmental factors, we made one-variate and multivariable logistic regression models. The results are presented in the form of ORs (odds ratios) with a 95% confidence interval (CI).

Table 3 shows the mean (SD, standard deviation) and median (quartiles) measurements of the working environment conditions in hospital premises during the study period. The mean air temperature in the hospital rooms was 23.19 °C, the mean air humidity was 32.88%, and the mean light intensity was 566.88 lux during the study period.

Table 4, Table 5 and Table 6 show the results of the univariable logistic regression model and the multivariable logistic regression model, determining the influence of the work environment factors on the probability of an incorrect result of a given parameter. The following environmental factors have been found to have a significant influence (*p* < 0.05) on the appearance of an abnormal tear meniscus result (≤0.2 mm).

In the univariable logistic regression model, for each additional 1 °C increase in temperature, the odds increased by 51.2%; an increase in relative air humidity (%) reduced these odds by 8.9%, and an increase in lighting by an additional 100 lux increased the risk by 12.3%.

In the multivariable regression model, each additional °C increased the odds by 2.4%, and as RH (relative humidity) increased, each additional percent reduced those odds by 0.48%. The risk of incorrect TMH measured results increased after work.

Each additional degree in Celsius increased these chances a little more than twofold, while each additional percentage increase in humidity reduced these chances by 4.3%. In the multivariable model, the air temperature had a significant impact on the risk of an abnormal TMH result after work. Each additional degree in Celsius increased this risk by 10.7%.

An independent predictor of the odds of an abnormal NIKBUT score (<10 s) measured before work was male gender, which reduced these odds by 26.2% compared to females. The chances of obtaining an incorrect result (NIKBUT < 10 s), measured after work, were significantly influenced by the lighting intensity, with each subsequent 100 lux reducing these chances by 12.3% and each subsequent year of life increasing the chances by 0.784%.

It was found that the risk of incorrect Schirmer test result < 10 mm in the OW and MW groups increased each additional degree in Celsius by 45.1%, and each additional 100 lux of lighting increased this risk by 31.5%. Each year of life increased these chances by 0.8%, working at a computer for over 4 h a day increased this risk by 18.6%, artificial lighting increased these chances by 21.5%, and each additional 100 lux increased the risk by 3.2%. For each additional degree in Celsius increase in air temperature, the risk increased by 3.8%. Each subsequent year of life increased the risk of Meibomian gland dysfunction (MGD) by 0.628% and working at a screen for over 4 h a day increased this risk by 18.1%—associated with reduced blink frequency, which causes excessive evaporation of the eye surface. Analyzing the factors influencing the risk of obtaining an erroneous result >12 points in the OSDI questionnaire, we found that age increased the risk by 1.3%, and work in air-conditioned rooms increased this risk to 25.5%.

An independent predictor of the appearance of DED diagnosis was influenced by the age of the respondents, increasing the risk by 0.782%.

### 3.5. Results after 1-Year Follow-Up

**Figure 7:**
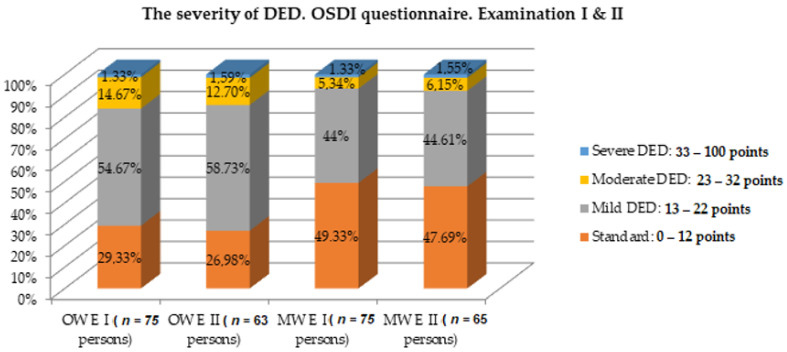
Comparison of the results of the OSDI questionnaire in both groups at the beginning of the study and after 1 year.

After 1 year, a control examination was performed in the OW group in 63 people (examination II) and in the MW group in 65 people. The OSDI questionnaire was also reverified. On the basis of the ocular surface disturbance index, we found that examination II showed no statistically significant difference in the severity of dry eye syndrome. The results are shown in Table 7 and Figure 7.

**Figure 8:**
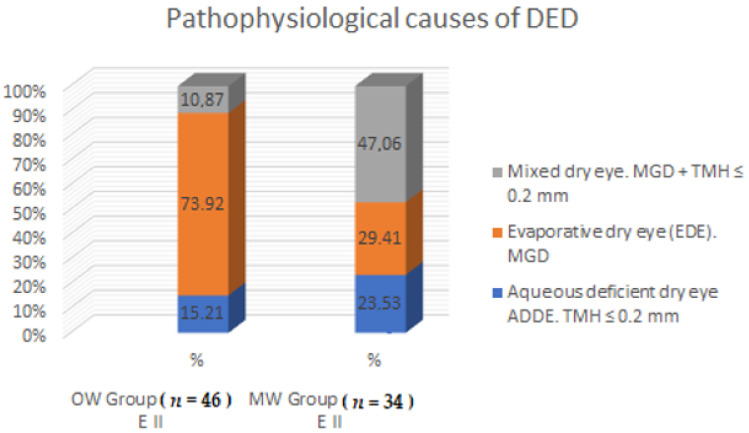
Pathophysiological causes of dry eye disease in the OW and MW groups at the 1-year follow-up.

There was a statistically significant difference (*p* < 0.05) in the occurrence of dry eye disease between the OW and MW groups at the 1-year follow-up. Dry eye disease in the OW group was present in 73.02% of people (*n* = 46), while in the MW group it occurred in 52.31% (*n* = 34) of the respondents (Table 7 and Figure 7). It has been shown that there is a significant statistical difference between the causes of dry eye disease in both groups (Table 8 and Figure 8).

**Figure 9:**
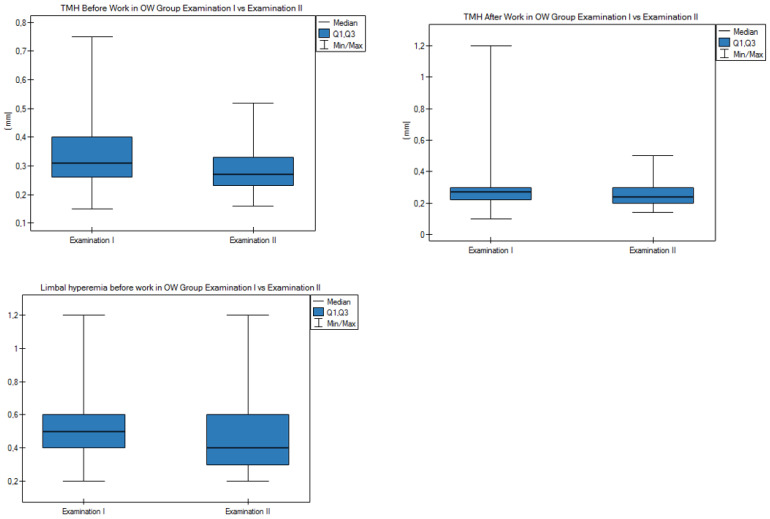
Measurement results of the eye surface parameters in the OW group and in the MW group at the 1-year follow-up. Table S5 is available in the Supplementary Materials.

At the 1-year follow-up, the eye surface parameters were re-measured using a keratograph to determine if there was a significant change in the eye surface parameters. The obtained results are presented in Table S5 (in the Supplementary Materials) and Figure 9.

In the OW group, there was a statistically significant (*p* < 0.05) difference for the mean TMH result before and after work and for mean conjunctival and limbal hyperemia before work. In the MW group, there was no statistically significant reduction in the mean TMH result before and after work. Statistically significant differences were observed in mean conjunctival hyperemia before work in the MW group. In the OW group in examination I (first/baseline examination), the mean TMH result before work was 0.34 mm vs. 0.28 mm at II (examination at 1-year follow-up); the mean result of the TMH after work in examination I was 0.28 mm vs. 0.25 mm for examination II. The mean result of conjunctival and limbal hyperemia before work in the OW examination I group was 0.73 and 0.52, respectively, vs. 0.69 and 0.47 for examination II. In examination II at the 1-year follow-up, these measurements were statistically significantly lower than in examination I. The mean result of the Schirmer test did not change significantly in both studied groups.

## 4. Discussion

In this study, we evaluated the effect of environmental factors on the symptoms and signs of dry eye disease in medical workers and office workers at the same hospital. We focused on the impact of working environment factors, such as relative air humidity and temperature, lighting, time spent at a computer, and the presence of air conditioning in the hospital workplace. Our results indicated that environmental factors might have resulted in ocular effects and differences in ocular surface parameters. The results of our work were based primarily on the objective assessment of the ocular surface parameters using the Keratograph 5M, but also on a subjective assessment using the OSDI questionnaire.

Kuo et al. mentioned in their recently published research that bilateral eyes of DED patients may have similar but different ocular surface performance and tear composition [28]. Similar to the research of other authors in our study results, we included the first examined eye because automatic measurement may produce distorted measurements and results from the second examined eye [28,30].

In the present study, subjective mild dry eye disease was found in 41 persons (54.67%) and moderate in 11 persons—14.67% more in the OW group than in the MW group (Figure 1). At the 1-year follow-up, no changes were observed. The severity of the dry eye syndrome in terms of the points obtained in the OSDI questionnaire in the OW and MW groups is presented in Table 7.

In the OW group, evaporative dry eye (EDE) (41 persons = 77.36%) was more frequent, while aqueous deficient dry eye (ADDE) and evaporative dry eye (EDE) were less frequent than in the MW group. In the MW group, the most common cause was mixed dry eye (Table 2). After 1 year, the most common cause of DED was EDE, which affected 34 persons in the OW group (73.92%), while in the MW group, the most common cause of DED was mixed dry eye (16 persons = 47.06%). A similar incidence of dry eye syndrome is described in other authors’ studies, in which the predominant cause of DED is EDE, similar to our study [31,32].

As was the case for other authors, in our study, there was a significant difference in the incidence of DED depending on the diagnostic criteria (subjective or objective) [7]. In our study, DED was diagnosed much more often on the basis of the OSDI questionnaire. Most studies indicate a higher diagnostic value and the advantage of objective tests over subjective tests [7].

Many authors emphasize the influence of female sex as a predisposing factor to DED [4,25]. According to the TFOS DEWS II report based on the OSDI index, women (33.6%) reported symptoms of dry eye disease more often than men (15.6%) [7]. Similarly, according to the SANDE (The Symptom Assessment in Dry Eye) questionnaire, dry eye disease was diagnosed much more often in women (39.1%) than in men (17.9%) [33]. The difference in the prevalence of DED in women is mainly due to the action of steroid hormones (androgens, estrogen, progesterone) on the surface of the eye [33]. After the age of 40, under the influence of hormonal changes and environmental and psychosocial factors, the tear film, the surface of the eye, and the lacrimal gland change [34]. In our study, there was no effect of gender on the incidence of DED, as no statistically significant difference was found between men and women in both groups. The reason for the lack of such a relationship may be the fact that, in both groups, there were fewer men than women—67 women (89.33%) and 8 men (10.67%) in the OW group vs. 63 women (84%) and 12 men (16%) in the MW group—which is a limitation of our study.

It is well known, and has been emphasized many times in the literature, that the older the person, the greater the probability of dry eye disease [4,6,25]. In our study, we showed that in the group of people over 55 years of age, dry eye was diagnosed in 62.86%. Moreover, each subsequent year of life increases the risk of developing dry eye disease by 0.782%, as shown in Table 5 and Table 6. Other authors have reported that women are on average 6 years younger than men at the time of diagnosis, with the mean age for women being 60 years vs. 66 years for men [33].

In our study, the groups differed statistically significantly (*p* ≤ 0.05) in terms of the mean TMH result before and after work, as well as the mean time of NIKBUT result after work. In the OW and MW groups, a statistically significant difference was demonstrated for the mean results of TMH measurements before and after work, being lower after work in both groups (Table 5 and Figure 5). In the OW group, it was found that, at the 1-year follow-up, the mean TMH result was statistically significantly lower than in study I (Table S5 and Figure 9). In both the OW and MW groups, a decrease in the mean TMH result after work was observed, which may be related to the time of day; however, in the logistic regression model, it was found that the risk of abnormal TMH results was influenced by the relative humidity and air temperature. The results are shown in Table 5, Table 6 and Table 7.

The groups presented statistically significant differences for the mean NIKBUT result after work—NIKBUT in the OW group was 11.18 s vs. 12.53 s in the MW group (Table S1). Comparing the results before and after work in both groups, the mean NIKBUT result after work was statistically significantly shorter than before work (Table S3 and Figure 5). The obtained before–after work difference may be related to the influence of environmental factors or the time of day [35]. In the multivariable logistic regression model, it was found that the risk of an incorrect NIKBUT result (<10 s) after work was influenced by the illuminance—an increase of 100 lux reduced these chances by 12.3%, as shown in Table 5, Table 6 and Table 7. Similar observations were made by Wolkoff et al. in their research, pointing to the thinning of the outer lipid layer of the tear film during the working day, which resulted in a reduction of the tear film break time [35]. The thickness of the tear film is highest shortly after waking up, but it fluctuates during the day and becomes thinner after a day of activity [35]. Smedbold et al. assessed the relationship between the environment and signs of eye irritation in nurses working in different departments of geriatric hospitals and revealed that, in high-temperature environments with low humidity, the tear evaporation rate was increased, causing instability of the tear film [36,37]. In a study by Castellanos-González and co-authors assessing the prevalence of dry eye disease in residents of various surgical specialties, 56% of physicians who were exposed to irritating environmental factors in the workplace were found to have dry eye disease on the basis of the OSDI questionnaire [38].

The presence of air conditioning or ventilation in hospital rooms reduces the air humidity and thus contributes to excessive evaporation of the tear film. In our study, we found that there is a difference in the mean TMH result before work, where the result was higher for the OW group. There was also a statistically significant difference for the mean Schirmer’s test results in the OW group and in the MW group for people working with and without air conditioning (Table S3 and Figure 5). This may be related to evaporative dry eye (EDE) and aqueous deficient dry eye (ADDE) or mixed DED.

In people working in air-conditioned rooms, higher mean conjunctival hyperemia was observed in both groups but had no statistical significance. The mean results of limbal hyperemia before and after work in both groups were higher for people working in air-conditioned rooms. In the MW group, we found statistically significant differences for limbal hyperemia before and after work (Table S3 and Figure 5). Low relative air humidity in the building and air-conditioned rooms negatively affects the tear film, causing symptoms of DED [37]. In the multivariable logistic regression model determining the risk of a score in the OSDI questionnaire of ≥13 points, it was found that working in air-conditioned rooms increases the risk by 25.5%.

Our study showed that working at a computer negatively affects specific parameters of the eye surface. In the study, we compared the mean TMH value before and after work in both groups and found no statistically significant differences for the two groups. For people working at a computer for over 4 h in the OW group, the mean THM result was higher than in the group working at a computer for less than 4 h. Comparing the parameters for people working at a computer for up to 4 h, the mean TMH result was higher for the OW group before and after work compared to the MW group (Table S4 and Figure 6). Our study showed that in the OW group in people working >4 h at a computer, the mean NIKBUT result before work and the mean Schirmer test result were statistically significantly lower than in people working at a computer for less than 4 h. Uchino et al. and Castellanos-González et al., similar to in our study, demonstrated the shortening of NIKBUT for people working with a computer, while the Schirmer test result in this study did not change significantly [25,37]. In both groups, we found lowered mean values of the height of the tear meniscus and NIKBUT before and after work in people working at a computer for more than 4 h. This could be caused by prolonged pauses in blinking when viewing a screen. In studies by other authors, it was found that working with devices with a screen increases the percentage of incomplete blinks and accelerates the evaporation of the tear film [26,37,38]. The results of measurements of the degree of conjunctival hyperemia before work were statistically significantly higher for people working at a computer for more than 4 h a day in both groups as well as after work in the MW group. There were statistically significant differences for the mean limbal hyperemia results in the OW group before work. The mean conjunctiva and limbus hyperemia results were higher in people working at a computer for more than 4 h. The study showed a significant connection between working at a screen ≥ 4 h a day and the occurrence of Meibomian gland dysfunction (MGD). People working at a computer for 4 or more hours a day were more likely to have MGD compared to those working at a computer for less than 4 h a day. On the basis of the multivariable logistic regression, we found that working at a computer for over 4 h a day increased the chances of MGD by 18.1% (Table 5 and Table 6).

Indoor environmental contamination also has adverse health effects. Environmental irritants can be associated with “sick building syndrome” (i.e., a disease caused by irritants found in the workplace, volatile organic compounds, and low-humidity conditions), in which there is an eye and mucous membrane irritation, causing an unstable tear film in office workers [39,40]. In 1992, Norn stated that “sick building” workers have “pollution keratoconjunctivitis” with a decline in BUT (break-up time) values and epithelial alterations detected by lissamine green staining [41].

In our study, we assessed the effects of air temperature, relative air humidity, and lighting on ocular surface parameters. The median (quartiles) air temperature was 23 °C (22–24 °C), the median relative air humidity was 31% (28–37.75%), and the median lighting was 467 lux (370.35–600). Similar results for hospital rooms were obtained by Norwegian authors, with an air temperature of 23.2 °C (23–23.7 °C) and a relative air humidity of 24% (17–26%) [36]. We have shown that the risk of an incorrect Schirmer test result increased by 21.5% in people staying in rooms with an artificial light source. We have also shown that excessive lighting affects the NIKBUT result, which, when increased by an additional 100 lux, reduces these chances by 12.3%, and an abnormal THM result before work is affected by each additional 100 lux, which increases the risk by 12.3%. Numerous epidemiological studies have shown a strong connection between reduced relative air humidity, temperature rise, time of day, poor lighting, and the presence of symptoms of dry eye disease, which we also observed in our study [25,34,42,43]. The TMH depends on the temperature and humidity of the air, but also on the time of day, air velocity, and lighting [25]. Increasing the relative humidity by an additional percent reduces the risk of incorrect results by 8.9% in the univariable logistic regression model, while in a multivariable model this risk is reduced by 0.48%. Every additional degree in Celsius increase in air temperature increases the risk of an abnormal THM result before work by 51.2%, and post-work tear meniscus height doubles the risk. Other studies indicate that excessive evaporation of the tear film increases when the relative air humidity is 5–70%; with an increase in air humidity of over 70%, excessive evaporation is reduced to 0 [44]. We demonstrated in our research that an incorrect Schirmer test result was influenced by the temperature and relative air humidity. Every degree in Celsius increase in temperature increases the risk of incorrect results by 45.1%, and each additional percent increase in humidity reduces the risk of an incorrect Schirmer test result by 3.8%, showing that a reduced relative air humidity causes more frequent blinking and results in eye discomfort [35].

The study is not without limitations. The authors are aware that not all tests for the diagnosis of dry eye disease recommended by TFOS DEWS II have been performed according to the latest guidelines, such as the measurement of tear film osmolarity or eye surface staining, which should be included in future research. One justification may be the fact that the current study focused primarily on simple and noninvasive methods for measuring the parameters of the eye surface, which are currently recommended in the diagnosis of DED [25].

As with all studies, our results must be considered in light of their limitations. The limitation of a study assessing the influence of environmental factors in hospital rooms is that it is impossible to eliminate the influence of other factors that affect the eye surface in office workers and medical workers outside working hours, including lifestyle, genetic conditions, individual predispositions, socioeconomic status, and exposure to stress. Creation of ideal experimental conditions in hospital rooms, as well as exposure to these factors, is difficult, because the test subjects performed their work in various places and were exposed to other environmental factors such as relative humidity, air temperature, and lighting intensity. The isolation of individual factors and the assessment of their impact on the individual parameters of the eye surface is impossible in hospital rooms, except for conditions that can be strictly controlled in laboratory conditions. Nevertheless, we attempted to examine the impact of the actual environmental conditions of the daily work of medical personnel, often with a variety of performed duties. Future studies are needed to more precisely evaluate the relationships between working environmental conditions and conditions such as lifestyle and living environment and their influence on the ocular surface. Perhaps modifying environmental factors can help us to determine which of them are most beneficial for modifying the disease status and can guide the therapeutic strategy in DED.

## 5. Conclusions

Environmental factors such as reduced relative air humidity, increased air temperature, and decreased illumination have a negative impact on the ocular surface; therefore, an effort should be made to ensure air quality, proper room temperature, and adequate illumination in the work environment. DED eye drops should be designed to target ocular surface abnormalities according to their etiology. Inflammation proved to be a significant component of DED, caused or exaggerated by air conditioning and working at a computer screen.

## Figures and Tables

**Table 1:** Comparison of demographic data at baseline.

Variables			OW Group (*n* = 75)			MW Group (*n* = 75)		OW + MW Groups (*n* = 150)			*p* *
			*n*	%		*n*	%	*n*		%	
Sex	F		67	89.33%		63	84.00%	130		86.67%	0.471
	M		8	10.67%		12	16.00%	20		13.33%	
Variables	OW Group (*N* = 75)		MW Group (*n* = 75)				OW + MW Groups (*n* = 150)				*p* **
	Mean (SD)	Median (quartiles)	Mean (SD)		Median (quartiles)		Mean (SD)		Median (quartiles)		
Age (years)	47.0 (8.4)	47.0 (40.0–54.0)	47.1 (12.8)		49.0 (38.5–56.5)		47.1 (10.8)		48.0 (40.0–55.0)		0.555
OSDI (score)	15.2 (6.3)	15 (12.0–18.0)	11.8 (7.3)		13 (7.5–1.5)		13.5 (7.0)		14 (9.0–17.0)		0.002

* Chi-squared test; F = Fisher’s exact test (low values expected in the table), ** Mann-Whitney test (due to non-normal distribution). Legend: OSDI—index of eye surface disorders, OW group—office workers group, MW group—medical workers group.

**Table 2:** Pathophysiological causes of dry eye syndrome.

Pathophysiological Causes	OW Group (*n* = 53)		MW Group (*n* = 38)		*p* *
	*n*	%	*n*	%	
Aqueous deficient dry eye (ADDE), TMH ≤ 0.2 mm	6	11.32	8	21.05	0.001
Evaporative dry eye (EDE), MGD	41	77.36	12	31.58	
Mixed dry eye, MGD + TMH ≤ 0.2 mm	6	11.32	18	47.37	

* Fisher’s exact test (low values expected in the table); legend: OW group—office workers group, MW group—medical workers group.

**Table 3:** Results of measurements of temperature, relative air humidity, and lighting intensity in hospital rooms during the study period.

Variables	Mean (SD)	Median (Quartiles)
Air temperature (°C)	23.19 (1.47)	23 (22–24)
Relative air humidity (%)	32.88 (6.93)	31 (28–37.75)
Illumination (lux)	566.88 (314.58)	467 (370.25–600)

**Table 4:** Univariable logistic regression to determine the influence of temperature, relative air humidity, and lighting intensity on the incorrect results of the eye surface parameters.

	TMH (≤0.2 mm) Before Work		TMH (≤0.2 mm) After Work		NIKBUT (<10 s) Before Work		NIKBUT (<10 s) After Work		Schirmer Test (<10 mm)	
Variables	OR (95%CI)	*p* *	OR (95%CI)	*p* *	OR (95%CI)	*p* *	OR (95%CI)	*p* *	OR (95%CI)	*p* *
Air temperature (°C)	1.512 (1.1–2)	0	2.037 (1.6–2,6)	<0.001	0.856 (0.73–1.01)	0.06	0.889 (0.8–1)	0.1	1.451 (1.209–1.17)	<0.001
Relative air humidity (%)	0.911 (0.09–1)	0	0.957 (0.9–1)	0.04	1.024 (0.99–1.06)	0.18	0.975 (0.9–1)	0.1	0.972 (0.937–1)	0.135
Lighting intensity (lux)	1.123 (1.000–1.200)	0	1.064 (1–1.2)	0.12	0.928 (0.85–1.02)	0.11	0.877 (0.8–1)	0	1.315 (1.181–1.5)	<0.001

Legend: TMH—tear meniscus height; NIKBUT—non-invasive keratograph break-up time.

**Table 5:** Multivariable logistic regression to determine the influence of temperature, relative air humidity, and lighting intensity on the incorrect results of the eye surface parameters.

		TMH (≤0.2 mm) Before Work		TMH (≤0.2 mm) After Work		NIKBUT (<10 s) Before Work		NIKBUT (<10 s) After Work	
Variable		OR (95%CI)	*p*	OR (95%CI)	*p*	OR (95%CI)	*p*	OR (95%CI)	*p*
Air temperature (°C)		1.024 (1.01–1.04)	0.03	1.107 (1.073–1.142)	**<0.001**	0.964 (0.928–1.001)	0.057	0.977 (0.939–1.015)	0.234
Relative air humidity (%)		0.995 (0.99–0.99)	0.05	0.995 (0.989–1.002	0.192	1.004 (0.996–1.012)	0.357	0.992 (0.984–1.001)	0.067
Lighting intensity (lux)		1.008 (0.99–1.02)	0.23	0.997 (0.989–1.016	0.744	1.003 (0.981–1.026)	0.806	0.987 0.965–1.010)	0.273
Age		1.002 (0.99–1)	0.32	0.998 (0.992–1.004)	0.583	1.004 (0.997–1.011)	0.295	1.008 (1.000–1.015)	**0.037**
Working in an air-conditioned room	No	Reference	–	Reference	–	Reference	–	Reference	–
	Yes	1.011 (0.92–1.1)	0.8	1.076 (0.952–1.216)	0.242	0.965 (0.833–1.119)	0.638		
Time working at a computer	<4 h	Reference	–	Reference	–	Reference	–	Reference	–
	>4 h	1.024 (0.95–1.09)	0.5	0.982 (0.891–1.083)	0.716	1.092 (0.971–1.229)	0.144	1.06 (0.940–1.196)	0.344
Sex	F	Reference	–	Reference	–	Reference	–	Reference	–
	M	1.014 (0.88–1.16)	0.84	1.168 (0.962–1.419)	0.118	0.738 (0.583–0.933)	**0.012**	0.807 (0.645–1.026)	0.082
Lighting	Natural	Reference	–	Reference	–	Reference	–	Reference	–
	Artificial	0.992 (0.91–1.07)	0.86	0.953 (0.845–1.075)	0.432	1.034 (0.894–1.196)	0.652	0.974 (0.839–1.130)	0.726

OR: odds ratio; ref.: reference; legend: TMH—tear meniscus height; NIKBUT—non-invasive keratograph break-up time; F—female; M—male, OSDI—index of eye surface disorders, MGD—Meibomian gland dysfunction. Bold numbers—result is statistically significant *p* > 0.05.

**Table 6:** Multivariable logistic regression to determine the influence of temperature, relative air humidity, and lighting intensity on the incorrect results of the eye surface parameters.

		MGD		OSDI		DRY EYE		Schirmer Test (<10 mm)	
Variable		OR (95%CI)	*p*	OR (95%CI)	*p*	OR	*p*	OR (95%CI)	*p*
Air temperature (°C)		1.003 (0.972–1.034)	0.865	1.001 (0.968–1.035)	0.943	0.992 (0.954–1.033)	0.706	1.038 (1.005–1.071)	**0.024**
Relative air humidity (%)		0.99954 (0.993–1.006)	0.892	0.998 (0.991–1.005)	0.537	1.001 (0.993–1.01)	0.75	1.000 (0.993–1.007)	0.949
Lighting intensity (lux)		1.00013 (0.982–1.018)	0.988	1.016 (0.996–1.036)	0.116	1.002 (0.979–1.026)	0.837	1.032 (1.013–1.051)	**0.001**
Age		1.00628 (1.000–1.012)	**0.035**	1.013 (1.007–1.02)	**<0.001**	1.00782 (1.0028–101542)	**0.043**	1.008 (1.002–1.014)	**0.013**
Working in an air-conditioned room	No	Reference	–	Reference	–	Reference	–	Reference	–
	Yes	0.932 (0.827–1.05)	0.246	1.255 (1.103–1.428)	**0.001**	1.119 (0.959–1.307)	0.155	1.097 (0.969–1.242)	0.144
Time working at a computer	<4 h	Reference	–	Reference	–	Reference	–	Reference	–
	>4 h	1.181 (1.074–1.299)	**0.001**	1.058 (0.954–1.173)	0.285	1.103 (0.975–1.249)	0.121	1.186 (1.074–1.130)	**0.001**
Sex	F	Reference	–	Reference	–	Reference	–	Reference	–
	M	1.014 (0.838–1.227)	0.886	1.193 (0.971–1.466)	0.094	0.865 (0.676–1.107)	0.25	1.169 (0.960–1.425)	0.121
Lighting	Natural	Reference	–	Reference	–	Reference	–	Reference	–
	Artificial	1.112 (0.989–1.251)	0.078	1.052 (0.926–1.195)	0.437	0.967 (0.830–1.126)	0.663	1.215 (1.076–1.373)	**0.002**

OR: odds ratio; ref.: reference; legend: TMH—tear meniscus height; NIKBUT—non-invasive keratograph break-up time; F—female; M—male, OSDI—index of eye surface disorders, MGD—Meibomian gland dysfunction. Bold numbers—result is statistically significant *p* > 0.05.

**Table 7:** Comparison of the results of the OSDI questionnaire in both groups at the beginning of the study and after 1 year.

The Severity of DED OSDI Questionnaire	OW Group					MW Group				
	E I (*n* = 75)		E II (*n* = 63)		*p* *	E I (*n* = 75)		E II (*n* = 65)		*p* *
	*N*	%	*n*	%		*n*	%	*n*	%	
Standard: 0–12 points	22	29.33	17	26.98	0.948	37	49.33	31	47.69	0.783
Mild DED: 13–22 points	41	54.67	37	58.73		33	44	29	44.61	
Moderate DED: 23–32 points	11	14.67	8	12.7		4	5.34	4	6.15	
Severe DED: 33–100 points	1	1.33	1	1.59		1	1.33	1	1.55	

* Fisher’s exact test (low values expected in the table); legend: OW group—office workers group, MW group—medical workers group, DED—dry eye disease, E I—examination I at the beginning; E II—examination at 1-year follow-up.

**Table 8:** Pathophysiological causes of dry eye disease in the OW and MW groups at the 1-year follow-up.

Pathophysiological Causes of DED	OW Group (*n* = 46); E II		MW Group (*n* = 34); E II		*p* *
	*N*	%	*n*	%	
Aqueous deficient dry eye (ADDE), TMH ≤ 0.2 mm	7	15.21	8	23.53	0.001
Evaporative dry eye (EDE), MGD	34	73.92	10	29.41	
Mixed dry eye, MGD + TMH ≤ 0.2 mm	5	10.87	16	47.06	

* Fisher’s exact test (low values expected in the table); legend: DED—dry eye disease, OW group—office workers group, MW group—medical workers group; E II—examination after 1-year follow-up.

## Data Availability

Data available on request due to restrictions.

## Supplementary Materials

The following are available online at https://www.mdpi.com/2075-4418/11/3/392/s1: Table S1: Comparison of the measurement results of the eye surface parameters in the OW and MW groups. Table S2: Measurement results of the eye surface parameters measured “before–after” work in the OW and MW groups. Table S3: Comparison of the results of the parameters of the eye surface in the OW and MW groups staying in air-conditioned rooms and rooms without conditioning. Table S4: Working time at the computer, and the results of measurements of the eye surface parameters in the OW and MW groups. Table S5: Measurement results of the eye surface parameters in the OW and MW groups after 1-year follow-up.

## Abbreviations

TFOS	Tear Film and Ocular Surface Society
ADDE	Aqueous deficient dry eye
AC	Air conditioning
CI	Confidence interval
DED	Dry eye disease
DEWS	Dry eye workshop
EDE	Evaporative dry eye
LASIK	Laser-assisted in situ keratomileusis
MGD	Meibomian gland dysfunction
MW	Medical workers
NIKBUT	Non-invasive keratographic break-up time
Non-AC	Non-air-conditioned
ORs	Odds ratios
OSDI	Ocular Surface Disease Index
OW	Office workers
RH	Relative humidity
SANDE	The Symptom Assessment in Dry Eye
SD	Standard deviation
TMH	Tear meniscus height
VDT	Video display terminals
