# Supplementary material for: The Influence of Work Environment Factors on the Ocular Surface in a One-Year Follow-Up Prospective Clinical Study
Source: Diagnostics (Basel). 2021 Feb 25;11(3):392. doi: 10.3390/diagnostics11030392 (PMC7996489; doi:10.3390/diagnostics11030392)
Supplement: Supplementary file 1 [file diagnostics-11-00392-s001.zip › diagnostics-1081000-supplementary.pdf]

Table S1. Comparison of the measurement results of the eye surface parameters in the OW and MW groups.

Variables	Group	N=150 eyes	Mean	SD	Median	Min	Max	Q1	Q3	p *
TMH before work [mm]	OW	75	0.34	0.01	0.31	0.15	0.75	0.26	0.4	0.004
	MW	75	0.29	0.01	0.27	0.12	0.75	0.23	0.325	
TMH after work [mm]	OW	75	0.29	0.02	0.27	0.1	1.2	0.22	0.3	0.007
	MW	75	0.26	0.10	0.26	0.10	0.90	0.19	0.29	
NIKBUT (first) before work[s]	OW	75	12.43	6.33	11.34	2.21	24.15	7.12	17.15	0.135
	MW	75	14.11	5.61	13.83	2.71	24.09	10.43	18.56	
NIKBUT (first) after work [s]	OW	75	11.18	6.33	9.43	2.42	23.56	6.15	16.70	0.009
	MW	75	12.53	5.19	12.05	3.19	24.00	9.06	16.02	
Conjunctival hyperemia before work	OW	75	0.73	0.22	0.70	0.30	1.50	0.60	0.80	0.300
	MW	75	0.71	0.34	0.60	0.20	1.60	0.40	0.90	
Conjunctival hyperemia after work	OW	75	0.72	0.29	0.70	0.30	2.20	0.50	0.80	0.215
	MW	75	0.67	0.32	0.60	0.20	1.50	0.40	0.80	
Limbal hyperemiabefore work	OW	75	0.52	0.23	0.50	0.20	1.20	0.40	0.60	0.284
	MW	75	0.52	0.29	0.40	0.20	1.70	0.30	0.60	
Limbal hyperemia after work	OW	75	0.50	0.24	0.50	0.20	1.70	0.30	0.60	0.997
	MW	75	0.51	0.24	0.40	0.20	1.20	0.40	0.60	
Schirmer test [mm]	OW	75	12.05	3.70	12.00	6.00	21.00	9.00	14.00	0.495
	MW	75	12.84	4.74	12.00	5.00	26.00	10.00	15.00	

MGD	**OW Group (N=75 eyes)**		**MW Group (N=75 eyes)**		**p ****
	n	%	n	%	
No	60	80%	61	81.34%	1
Yes	15	20%	14	18.66%	

*Mann – Whitney test ** Chi–square test; Legend: TMH – tear meniscus height; NIKBUT–Non–Invasive Keratograph Break–Up Time; MGD – Meibomian Gland Dysfunction

Table S2. Measurement results of the eye surface parameters measured "before-after" work in the OW group and in the MW group.

Variables	Group	Time	Mean	SD	Median	Min	Max	Q1	Q3	p *
TMH [mm]	OW	Before work	0.34	0.01	0.31	0.15	0.75	0.26	0.4	<0.001
		After work	0.29	0.02	0.27	0.1	1.2	0.22	0.3	
	MW	Before work	0.29	0.01	0.27	0.12	0.75	0.23	0.325	<0.001
		After work	0.26	0.10	0.26	0.10	0.90	0.19	0.29	
NIKBUT [first]	OW	Before work	12.43	6.33	11.34	2.21	24.15	7.12	17.15	<0.001
		After work	11.18	6.33	9.43	2.42	23.56	6.15	16.70	
	MW	Before work	14.11	5.61	13.83	2.71	24.09	10.43	18.56	0.003
		After work	12.53	5.19	12.05	3.19	24.00	9.06	16.02	
Conjunctival hyperemia	OW	Before work	0.73	0.22	0.70	0.30	1.50	0.60	0.80	0.652
		After work	0.72	0.29	0.70	0.30	2.20	0.50	0.80	
	MW	Before work	0.71	0.34	0.60	0.20	1.60	0.40	0.90	0.03
		After work	0.67	0.32	0.60	0.20	1.50	0.40	0.80	
Limbal hyperemia	OW	Before work	0.52	0.23	0.50	0.20	1.20	0.40	0.60	0.314
		After work	0.50	0.24	0.50	0.20	1.70	0.30	0.60	
	MW	Before work	0.52	0.29	0.40	0.20	1.70	0.30	0.60	0.961
		After work	0.51	0.24	0.40	0.20	1.20	0.40	0.60	

Table S3. Comparison of the results of the parameters of the eye surface in the OW group and in the MW group staying in air-conditioned rooms and rooms without conditioning.

Parametrer	G	Air-conditioned	N=150 eyes	MEAN	SD	Mediana	Min	Max	Q1	Q3	p *
TMH before work [mm]	OW	Yes	51	0.32	0.11	0.3	0.15	0.7	0.26	0.35	0.375
		No	24	0.39	0.14	0.35	0.24	0.75	0.27	0.46	
	MW	Yes	28	0.28	0.1	0.26	0.12	0.63	0.22	0.32	0.331
		No	47	0.29	0.09	0.27	0.13	0.75	0.23	0.32	
TMH after work [mm]	OW	Yes	51	0.26	0.07	0.26	0.1	0.5	0.22	0.3	0.489
		No	24	0.37	0.25	0.28	0.14	1.2	0.24	0.39	
	MW	Yes	28	0.25	0.08	0.24	0.1	0.45	0.19	0.3	0.647
		No	47	0.26	0.12	0.26	0.13	0.9	0.2	0.28	
NIKBUT (first) before work [s]	OW	Yes	51	12.68	5.59	11.39	2.21	24.15	6.78	18.04	0.144
		No	24	11.33	5.56	9.87	3.49	23.21	7.5	14.61	
	MW	Yes	28	13.25	4.63	13.63	2.8	20.99	10.29	17.24	0.308
		No	47	14.61	6.11	14.44	2.71	24	10.57	19.22	
NIKBUT (first) after work [s]	OW	Yes	51	11.72	6.24	10	46	23.56	6.46	17.05	0.596
		No	24	9.14	6.06	7.24	2.42	23.12	5.2	9.3	
	MW	Yes	28	12.04	3.65	11.86	5.02	20.34	9.34	14.14	0.679
		No	47	12.87	5.82	11.65	3.19	24	8.81	16.51	
Conjunctival hyperemia before work	OW	Yes	51	0.73	0.19	0.7	0.4	1.3	0.6	0.9	0.542
		No	24	0.69	0.3	0.65	0.3	1.5	0.5	0.7	
	MW	Yes	28	0.67	0.32	0.6	0.3	1.5	0.4	0.8	0.360
		No	47	0.73	0.34	0.7	0.2	1.6	0.5	1	
Conjunctival hyperemia after work	OW	Yes	51	0.73	0.31	0.7	0.3	2.2	0.5	0.8	0.855
		No	24	0.64	0.21	0.65	0.3	1	0.47	0.8	
	MW	Yes	28	0.64	0.28	0.6	0.3	1.4	0.4	0.8	0.693
		No	47	0.68	0.33	0.6	0.2	1.5	0.4	0.8	
Limbal hyperemia before work	OW	Yes	51	0.52	0.23	0.5	0.2	1.2	0.4	0.7	0.424
		No	24	0.51	0.22	0.5	0.2	1.1	0.4	0.6	
	MW	Yes	28	0.47	0.28	0.4	0.2	1.5	0.3	0.5	0.016
		No	47	0.54	0.3	0.4	0.2	1.3	0.3	0.65	
Limbal hyperemia after work	OW	Yes	51	0.51	0.24	0.5	0.2	1.7	0.4	0.6	0.339
		No	24	0.44	0.23	0.4	0.2	1.1	0.3	0.6	
	MW	Yes	28	0.47	0.22	0.4	0.2	1.2	0.3	0.5	0.014
		No	47	0.53	0.24	0.4	0.3	1.2	0.4	0.6	
Schirmer test [mm]	OW	Yes	51	11.35	3.38	11.43	6	21	9	14	<0.002
		No	24	14.75	3.62	15.5	8	21	12.75	17	
	MW	Yes	28	11.6	3.65	12	6	22	9.75	14	0.015
		No	47	13.57	5.17	13	5	26	10	16	

* Test Manna-Whitney'a; Legend: TMH – tear meniscus height; NIKBUT-Non-Invasive Keratograph Break-Up Time; OW Group – Office Workers Group, MW Group – Medical Workers Group, G-Group

Table S4. Working time at the computer, and the results of measurements of the eye surface parameters in the OW group and in the MW group.

Variables		Working time at the computer	N=150 eyes	Mean	SD	Median	Min	Max	Q1	Q3	p *
**TMH before work [mm]**	OW	<4h	34	0.35	0.12	0.31	0.15	0.70	0.27	0.40	0.893
		>4h	41	0.33	0.11	0.32	0.15	0.75	0.28	0.36	
	MW	<4h	52	0.28	0.12	0.24	0.12	0.75	0.21	0.32	0.401
		>4h	23	0.30	0.12	0.30	0.13	0.63	0.25	0.31	
**TMH after work [mm]**	OW	<4h	34	0.31	0.74	0.30	0.19	0.50	0.26	0.33	0.052
		>4h	41	0.26	0.10	0.26	0.10	0.70	0.20	0.30	
	MW	<4h	52	0.26	0.15	0.25	0.10	0.90	0.19	0.28	0.121
		>4h	23	0.29	0.09	0.29	0.15	0.45	0.24	0.35	
**NIKBUT (first) before work [s]**	OW	<4h	34	14.06	5.97	13.66	5.80	24.15	9.44	19.21	0.018
		>4h	41	11.05	5.93	9.03	3.49	24.00	6.70	14.29	
	MW	<4h	52	13.05	4.31	13.24	4.08	19.21	10.68	16.24	0.940
		>4h	23	13.20	4.48	13.34	2.80	21.00	12.09	14.24	
**NIKBUT (first) after work [s]**	OW	<4h	34	11.94	6.07	10.45	3.53	21.62	7.52	17.92	0.562
		>4h	41	11.06	6.58	8.90	2.46	23.56	6.33	16.28	
	MW	<4h	52	11.49	3.12	11.23	5.69	16.78	9.08	13.11	0.438
		>4h	23	12.08	4.56	12.59	3.19	18.34	9.67	15.45	
**Conjunctival hyperemia before work**	OW	<4h	34	0.72	0.19	0.70	0.30	1.30	0.60	0.80	0.035
		>4h	41	0.68	0.19	0.60	0.40	1.10	0.60	0.80	
	MW	<4h	52	0.62	0.30	0.50	0.30	1.50	0.40	0.80	0.006
		>4h	23	0.75	0.47	0.50	0.20	1.50	0.40	1.20	
**Conjunctival hyperemia after work**	OW	<4h	34	0.69	0.30	0.60	0.30	1.50	0.50	0.70	0.547
		>4h	41	0.71	0.31	0.70	0.30	2.20	0.50	0.80	
	MW	<4h	52	0.64	0.32	0.50	0.30	1.50	0.40	0.80	0.038
		>4h	23	0.69	0.41	0.50	0.30	1.50	0.40	1.10	
**Limbal hyperemia before work**	OW	<4h	34	0.58	0.22	0.60	0.30	1.20	0.40	0.70	0.017
		>4h	41	0.49	0.20	0.40	0.20	1.00	0.40	0.60	

	MW	<4h	52	0.41	0.17	0.40	0.20	0.90	0.30	0.40	0.087
		>4h	23	0.64	0.40	0.50	0.30	1.50	0.30	1.00	
**Limbal hyperemia after work**	OW	<4h	34	0.51	0.22	0.50	0.20	1.10	0.30	0.60	0.546
		>4h	41	0.49	0.26	0.40	0.20	1.70	0.38	0.60	
	MW	<4h	52	0.45	0.19	0.45	0.20	1.00	0.40	0.50	0.647
		>4h	23	0.58	0.35	0.40	0.30	1.20	0.30	0.90	
**Schirmer test [mm]**	OW	<4h	34	13.33	3.48	13.00	7.00	21.00	10.00	16.00	0.007
		>4h	41	10.80	3.19	10.00	6.00	19.00	8.00	13.00	
	MW	<4h	52	10.28	2.80	10.00	6.00	16.00	9.00	12.00	0.234
		>4h	23	12.23	5.01	11.00	5.00	22.00	10.00	14.00	

*Mann-Whitney Test; Legend: TMH – tear meniscus height; NIKBUT-Non-Invasive Keratograph Break-Up Time; OW Group – Office Workers Group, MW Group – Medical Workers Group,

Tabele S5. Measurement results of the eye surface parameters in the OW group and in the MW group after 1-year follow-up.

	Group	Time of examiantion	N	Mean	SD	Median	Min	Max	Q1	Q3	P *
**TMH before work [mm]**	OW	I	75	0.34	0.01	0.31	0.15	0.75	0.26	0.40	0.000
		II	63	0.28	0.79	0.27	0.16	0.52	0.23	0.33	
	MW	I	75	0.29	0.01	0.27	0.12	0.75	0.23	0.33	0.471
		II	65	0.24	0.10	0.26	0.10	0.80	0.22	0.26	
**TMH after work [mm]**	OW	I	75	0.29	0.02	0.27	0.10	1.20	0.22	0.30	0.047
		II	63	0.25	0.07	0.24	0.14	0.50	0.20	0.30	
	MW	I	75	0.26	0.10	0.26	0.10	0.90	0.19	0.29	0.387
		II	65	0.24	0.09	0.20	0.10	0.80	0.12	0.25	
**NIKBUT (first) before work [s]**	OW	I	75	12.43	6.33	11.34	2.21	24.15	7.12	17.15	0.222
		II	63	12.74	5.15	12.74	2.68	24.00	9.56	16.21	
	MW	I	75	14.11	5.61	13.83	2.71	24.09	10.43	18.56	0.684
		II	65	14.12	5.10	13.23	2.10	23.81	10.00	18.30	
**NIKBUT (first) after work [s]**	OW	I	75	11.18	6.33	9.43	2.42	23.56	6.15	16.70	0.498
		II	63	11.54	4.77	10.24	2.45	24.00	8.67	14.02	
	MW	I	75	12.53	5.19	12.05	3.19	24.00	9.06	16.02	0.241
		II	65	11.71	5.11	10.20	2.84	17.60	7.40	14.27	
**Conjunctival hyperemia before work**	OW	I	75	0.73	0.22	0.70	0.30	1.50	0.60	0.80	0.030
		II	63	0.69	0.18	0.70	0.40	1.40	0.60	0.80	
	MW	I	75	0.71	0.34	0.60	0.20	1.60	0.40	0.90	0.342
		II	65	0.70	0.31	0.50	0.20	1.50	0.40	0.80	
**Conjunctival hyperemia after work**	OW	I	75	0.72	0.29	0.70	0.30	2.20	0.50	0.80	0.274
		II	63	0.73	0.18	0.70	0.30	1.50	0.60	0.90	
	MW	I	75	0.67	0.32	0.60	0.20	1.50	0.40	0.80	0.054
		II	65	0.70	0.32	0.60	0.20	1.50	0.30	0.70	
**Limbal hyperemia before work**	OW	I	75	0.52	0.23	0.50	0.20	1.20	0.40	0.60	0.057
		II	63	0.47	0.19	0.40	0.20	1.20	0.30	0.60	
	MW	I	75	0.52	0.29	0.40	0.20	1.70	0.30	0.60	0.287
		II	65	0.51	0.27	0.50	0.20	1.70	0.30	0.60	
**Limbal hyperemia after work**	OW	I	75	0.50	0.24	0.50	0.20	1.70	0.30	0.60	0.200
		II	63	0.46	0.16	0.40	0.20	1.00	0.40	0.60	
	MW	I	75	0.51	0.24	0.40	0.20	1.20	0.40	0.60	0.523

		II	65	0.50	0.22	0.40	0.20	1.00	0.40	0.50	
**Schirmer test [mm]**	OW	I	75	12.05	3.70	12.00	6.00	21.00	9.00	14.00	0.549
		II	63	12.45	4.33	12.00	5.00	26.00	9.00	14.00	
	MW	I	75	12.84	4.74	12.00	5.00	26.00	10.00	15.00	0.745
		II	65	12.23	4.75	12.00	5.00	25.80	9.00	14.00	

**MGD Before**	OW Group (N=75 eyes)		MW Group (N=75 eyes)		p **	MGD 1-year follow-up	OW Group (N=63 eyes)		MW Group (N=65 eyes)		p **
	n	%	n	%			n	%	n	%	
**No**	60	80.00%	59	78.67%	1	No	54	85.71%	54	83.07%	1
**Yes**	15	20.00%	16	21.33%		Yes	11	17.46%	11	16.93%	

* Wilcoxon test for dependent (repeated) measurements Legend: TMH- tear meniscus height, NIKBUT - non-invasive tear film break-up time, OW Group – Office Workers Group, MW Group – Medical Workers group E I – Examination I at the beginning; E II – Examination after 1-year follow-up.
